# Impact of pharmacist interventions on immunisation uptake: a systematic review and meta-analysis

**DOI:** 10.1080/20523211.2023.2285955

**Published:** 2023-12-07

**Authors:** Mohamad Hafiz Abd Rahim, Siti Hajar Mahamad Dom, Mohd Shah Rezan Hamzah, Siti Hawa Azman, Zahirah Zaharuddin, Mathumalar Loganathan Fahrni

**Affiliations:** a Faculty of Pharmacy, Universiti Teknologi MARA (UiTM) Puncak Alam Campus, Selangor Branch, Bandar Puncak Alam, Malaysia; b Center for Drug Policy and Health Economics Research (CDPHER), Universiti Teknologi MARA (UiTM), Puncak Alam, Malaysia

**Keywords:** Vaccination, primary prevention, immunisation, pharmacist, advocator

## Abstract

**Background:**

Under-utilisation of immunisation services remains a public health challenge. Pharmacists act as facilitators and increasingly as immunisers, yet relatively little robust evidence exists of the impact elicited on patient health outcome and vaccination uptake.

**Objective:**

To evaluate the influence of pharmacist interventions on public vaccination rate.

**Methods:**

SCOPUS, PubMed, and Web of Science were searched from inception to April 2023 to retrieve non- and randomised controlled clinical trials (RCTs). Studies were excluded if no comparator group to pharmacist involvement was reported. Data extraction, risk of bias assessments, and meta-analyses using random-effect models, were performed.

**Results:**

Four RCTs and 15 non-RCTs, encompassing influenza, pneumococcal, herpes zoster, and tetanus-diphtheria and pertussis vaccine types, and administered in diverse settings including community pharmacies, were included. Pooled effect sizes revealed that, as compared to usual care, pharmacists, regardless of their intervention, improved the overall immunisation uptake by up to 51% [RR 1.51 (1.28, 1.77)] while immunisation frequency doubled when pharmacists acted specifically as advocators [RR 2.09 (1.42, 3.07)].

**Conclusion:**

While the evidence for pharmacist immunisers was mixed, their contribution to immunisation programmes boosted public vaccination rate. Pharmacists demonstrated leadership and acquired indispensable advocator roles in the community and hospital settings. Future research could explore the depth of engagement and hence the extent of influence on immunisation uptake.

## Introduction

Immunisation is an essential component of preventive healthcare, with vaccines being widely recognised for their contribution in eliminating and mitigating a plethora of infectious diseases (Gagneur et al., 2019). Immunisation has several benefits, including reducing morbidity and mortality, lowering healthcare expenditures, and enhancing economic productivity (Sauer et al., 2021). The economic and health burden of vaccine-preventable diseases (VPDs) remain high worldwide, with developing countries bearing the brunt of their impact (DeStefano et al., 2019). According to the World Health Organization (WHO), VPDs accounted for approximately 1.5 million deaths each year, with children under the age of five years being the most vulnerable (DESA, 2019). Zhou et al. in 2022 estimated that the United States of America’s (USA) varicella vaccination programme generated a return on investment of $1.70 for every $1 spent during the first 25 years, with benefits continuing beyond 2020 (Zhou et al., 2022). The WHO also acknowledged and concurred that investing in immunisation programmes led to substantial savings in healthcare costs and increased economic productivity.

Despite the availability of safe and effective vaccines, many countries continue to experience suboptimal immunisation rates. Barriers to immunisation may have a significant impact on vaccination rates and consequently, to the incidence and prevalence of vaccine-preventable diseases. Healthcare professionals including pharmacists, can play an integral role in promoting immunisation uptake and enhancing public health outcomes (Brunelli et al., 2020). Given their expertise in medication, pharmacotherapy and pharmaceuticals, pharmacists are uniquely positioned to offer guidance and counselling on the benefits that outweigh the minimal risks associated with immunisation, and dispel myths and misconceptions regarding vaccines. With adequate training and exposure, pharmacists can then administer vaccines to patients (Haymarket, 2021). Additionally, pharmacists are also able to help identify those who qualify for vaccination, screen for contraindications and possible drug interactions, and offer follow-up care to ensure optimal vaccine safety and efficacy (Ibrahim et al., 2022).

Pharmacists across Europe are now authorised to optimise medication and dispense prescribed antibiotics and antiviral drugs to prevent infectious diseases. Additionally, in some countries, such as Argentina, the USA, Australia, France, Ireland, Italy, Norway, Poland, Portugal, Switzerland, and the United Kingdom (UK), pharmacists have been actively involved in vaccination programmes, and have demonstrated their potential to improve vaccination coverage (Buicu et al., 2018; Costa et al., 2017). In times of public health emergencies like during the recent COVID-19 pandemic, pharmacist immunisers in those nations were pivotal in ensuring the continuity and sustainability of vaccination services.

In the International Pharmaceutical Federation's 2016 global report, pharmacy impact on immunisation, and the training needs of the pharmacy workforce to successfully administer vaccines were outlined. Training contents included imparting skills on vaccine administration, knowledge of contra-indications and adverse reactions, anaphylaxis and cardio-pulmonary resuscitation, instructions, requirements and guidelines of local immunisation policies, and guidelines for the prevention of communicable diseases, including those induced by the Human Immunodeficiency Virus and Hepatitis viruses. Pharmacists are progressively acknowledged as valued members of the healthcare provider team who can improve immunisation uptake and other patient-related outcomes (Wubishet et al., 2021). In fact, in developed countries, pharmacists have progressively expanded their roles to become immunisation advocates and immunisers (Ciliberti et al., 2020). Pharmacist-led immunisation programmes were first introduced in Canada in 2012, when pharmacists were authorised to administer flu shots in Ontario (Houle et al., 2019). This was followed by the implementation of similar programmes in parts of theUK, Australia, and New Zealand (Aguilar et al., 2021).

The COVID-19 pandemic has highlighted the importance of involving pharmacists in non-clinical services, such as procurement, storage, and transportation, as well as clinical services, including reconstitution, dispensing, and administering vaccines to provide comprehensive pharmaceutical care to patients. There are several studies that had assessed the consequences of pharmacist involvement on immunisation programmes, and the results have been promising. Specifically, several investigations had explored the impact of pharmacy-based vaccine administration on influenza, pneumococcal, and herpes zoster vaccination rates. While earlier published reviews and meta-analyses indicated that pharmacist involvement in vaccine administration had a statistically significant effect on immunisation rates, many of the included studies utilised relatively weaker study designs, such as controlled before-after, retrospective cohort, and quasi-experimental, which exhibited a considerable amount of heterogeneity and could have potentially deflated the impact on immunisation rates. Many of the meta-analyses included a limited number of trials with high risks of biases or focused solely on individual intervention such as pharmacist as immunisers without a control group.

Hence, we set out to evaluate, the broader impact of pharmacist interventions on immunisation rates. Our objectives were twofold: to perform an up-to-date systematic review and meta-analysis by incorporating recent clinical and controlled trials, and to examine the influence of pharmacist participation, whether as an immuniser or an advocate, or both, on public vaccination rate. The reporting of this meta-analysis adhered to the Preferred Reporting Items for Systematic Reviews and Meta-Analyses (PRISMA-2020) guideline (Page et al., 2021).

## Methods

### Eligibility criteria

We included studies of randomised clinical or controlled trials (RCTs) and non-randomised controlled trials (non-RCTs) that had a comparison group. The primary outcome of interest was the number of participants vaccinated or for studies that had more than one vaccine type, the number of doses administered. Immunisation rates were defined as the number of individuals who received the vaccines divided by the target population.

The non-RCTs included were categorised as, before and after studies, and cohort or longitudinal studies with a minimum of two groups. The comparison group received usual care, which was defined as routine care or standard of care received by patients without any pharmacist involvement.

Articles were limited to the English language. Letters to the editor, commentaries, conference papers, reviewers, editorials, and abstracts were excluded.

### Search strategy

We identified relevant studies using the following steps. Firstly, we developed a comprehensive search strategy using Boolean search strings. We conducted a comprehensive systematic search of the SCOPUS, Web of Science (WOS), and PubMed databases, from inception to 28 April 2023, using search terms related to vaccination and pharmacist. For example, we combined (immunisation or vaccination or booster or inoculation) AND (pharmacist or pharmacy or drug store or dispenser or apothecarist or druggist or chemist or pharmaceutical care). This string defined the population of interest (i.e. individuals who received immunisation), the exposure (i.e. involvement of pharmacists in the immunisation process), and the outcomes of interest (i.e. the number of people vaccinated). The detailed search strategy and complete search string is included in the Supplementary Materials, Supplement I. We also examined the reference lists of previous systematic reviews to identify additional relevant studies. Duplicate articles identified from multiple sources were removed. Authors were contacted in an attempt to retrieve full texts or raw data that were not made available online.

### Data extraction

Titles and abstracts of all retrieved articles studies were screened independently by two reviewers. Full-text screening of each potential article was independently done by the reviewers (Z.Z and S.H.M) according to the eligibility criteria for inclusion in the systematic review and meta-analysis. To resolve any discrepancy, a third reviewer (M.S.R.H) was consulted before an agreement was reached. After the selection stage was completed, a standardised data extraction form was used to extract the data (S.H.A) and charting was performed.

The following details were charted from each study: author name, publication year, country, study design and setting. The pharmacists’ roles in the immunisation process were defined as (i) an immuniser or an advocator (defined as pharmacists who advocated immunisation through education, training, campaigning, recruitment, or stocking up vaccines). The intervention (the mechanism through which a pharmacist achieved his or her aim or that which facilitated the pharmacists’ roles in increasing vaccine uptake) was categorised as (i) technology-based monitoring system, (ii) state law regulations, or (iii) quality improvement programme or funding. In addition, types of immunisation, outcome of interest, i.e., number of vaccine recipients or number of doses administered (for different vaccine-types) following the intervention, the age group of the recipients and the total number of recipients in the intervention and control groups were recorded.

### Risk of bias assessment

To assess the risk of bias of both randomised clinical/controlled trials and non-RCTs included in this meta-analysis, we employed two widely recognised tools: the Critical Appraisal Skills Programme (CASP) tool and the Cochrane Risk of Bias (ROB) 2 tool. The utilisation of both tools enabled the thorough evaluation of the risk of bias and methodological quality of the studies included, hence strengthening the reliability and validity of the systematic review and meta-analysis.

For consistency and dependability, the risk of bias evaluation was carried out independently by two reviewers (S.H.A. and M.S.R.H) and any discrepancy was resolved via discussion. The outcome was taken into consideration when interpreting the study's findings, drawing inferences, and assessing the overall calibre of the evidence in the meta-analysis.

### Effect measures

For effect size measurement, information on the changes or differences in the immunisation uptake were extracted and risk ratios were calculated for further analysis. A risk ratio of more than one indicated that the involvement of pharmacists was associated with better immunisation uptake as compared to no pharmacist involvement and vice versa. The intention-to-treat principle was used to estimate the risk ratio.

### Synthesis methods

Studies that reported the outcomes of interest in terms of crude numbers or proportion of events were deemed sufficient for calculation of risk ratio and were included in the meta-analysis. The effect sizes in the form of risk ratios and the pooled effect size was presented in table forms as well as graphical representation in the form of a Forest plot.

### Statistical analysis

The meta-analysis was performed using Review Manager 5.4 (RevMan). An inverse variance method with random effect modelling was applied to the analysis for the estimation of a pooled risk ratio (RR). This method allowed variation between included studies that are estimating different, but related, intervention effects. We reported the RR of individual studies together with their 95% confidence intervals and effect sizes were considered significant when the *p-*value was ≤ 0.05.

### Heterogeneity

The between-trial heterogeneity was assessed using the inconsistency index value (*I*^2^ statistic). The value of of <25%, 25–50%, 51–75% and >75% was considered low, moderate, high and very high respectively (Baroy et al., 2016). A 95% confidence interval was used as the measurement of uncertainty and the value was significant when the *p*-value was ≤ 0.05 .

### Subgroup analyses

Subgroup analyses were performed for the categories ‘service settings' and ‘role of pharmacists in the immunisation programmes' with the exclusion of studies with a high risk of bias (critical risk and serious risk).

### Reporting bias assessment

Publication bias was assessed visually using a funnel plot asymmetry test.

## Results

### Study selection

**Figure 1.**
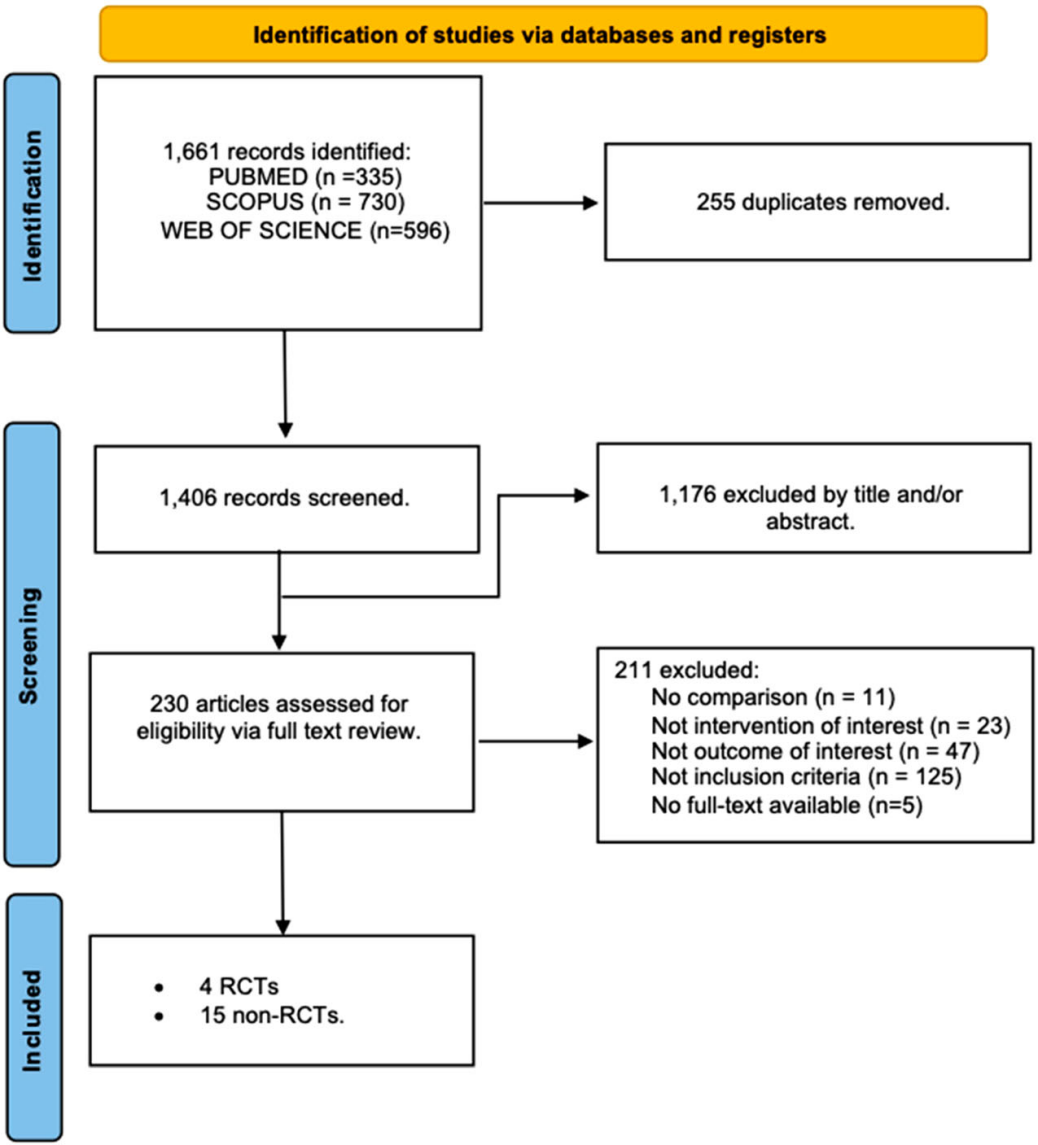
Preferred reporting items for systematic reviews and meta-analyses (PRISMA) flow chart for study selection. RCT, randomised controlled trial.

As demonstrated in Figure 1, a total of 1661 records were identified from the literature search. After the stages of title and abstract screening, and duplicate removal, 230 studies remained and were assessed for eligibility. We excluded a total of 211 studies for the following reasons: no comparison group (*n* = 11), did not study an intervention of interest (*n* = 23), did not study an outcome of interest (*n* = 47), did not satisfy the inclusion criteria (*n* = 125) or no full-text available (*n* = 5). The search yielded 4 RCTs and 15 non-RCTs.

### Study characteristics

Table 1 describes the characteristics of the included four RCTs studies (Heaton et al., 2022; Klassing et al., 2018; Ozdemir et al., 2023; Stolpe & Choudhry, 2019) and 15 non-RCTs studies (Abu-rish et al., 2021; Are et al., 2022; Bacci et al., 2019; Bayraktar-Ekincioglu et al., 2022; Cebollero et al., 2020; Deslandes et al., 2020; Fathima et al., 2021; Gatwood et al., 2022; Goldsworthy et al., 2022; Goode et al., 2023; Howe et al., 2022; Page et al., 2020; Rihtarchik et al., 2018; Scherrer et al., 2020; Villaverde Piñeiro et al., 2022). Two RCTs (Klassing et al., 2018; Stolpe & Choudhry, 2019) which were both conducted in the USA, evaluated the impact of technology-based systems that facilitated the advocacy strategies of pharmacists in identifying patients for vaccine recommendations. One RCT (Heaton et al., 2022), assessed two methods for pharmacists providing education on vaccines: either through phone calls or written letters to increase immunisation rates for influenza and pneumococcal vaccines in the USA. Another RCT (Ozdemir et al., 2023) conducted in Turkey evaluated the impact of face-to-face education on pneumococcal vaccines given by pharmacists to participants. These studies were conducted in two types of settings: community pharmacy (3 studies, 75%) (Heaton et al., 2022; Klassing et al., 2018; Stolpe & Choudhry, 2019) and hospital (1 study, 25%) (Ozdemir et al., 2023).

**Table 1.** Characteristics of studies included in the meta-analysis.

Author and year	Country	Study design	Setting	Pharmacists role	Intervention	Brief detail on intervention	Type(s) of immunisation	Outcome	Duration of interventional practice	Age group	*n*, intervention/control	Bias assessment
Abu-rish et al., 2021	Jordan	Non-RCT^a^	Hospital	Advocator^f^	Monitoring system^l^	Pharmacists discussed and provided booklet regarding the risk of the disease, the disease transmission, pneumococcal vaccines’ (PPSV23 and PCV13) indications, and possible side effects as per guideline recommendations. Comparison: Usual care	Pneumococcal	Number of vaccine recipients	2 months	≥65	683/844	Moderate risk
Are et al., 2022	United States	Non-RCT^b^	Community^c^	Immuniser	Monitoring system^k^	Pharmacists immunsation authority in different states that are categorised into 3 groups, independent authority, statewide protocol and collaborative practice agreement. Comparison: Without pharmacists immunisation authority	Influenza	Number of vaccine recipients	1 year	≥18	344,126/27,681	Critical risk
Bacci et al., 2019	United States	Non-RCT^a^	Community^c^	Advocator^i^/ Immuniser	Monitoring system^j^	A technology system, integrated in the pharmacies workflow that enable proactive checking of the immunisation information system and forecasting of and recommendation to patients about their vaccine needs. Comparison: Usual care	Influenza Pneumococcal Pertussis Herpes zoster	Number of vaccine doses administered	1 year	≥18	84,488/84,488	Moderate risk
Bayraktar-Ekincioglu et al., 2022	Turkey	Non-RCT^a^	Community^e^	Advocator^f^	Monitoring system^l^	Pharmacists provide education to participants regarding vaccines. Comparison: Usual care	Pneumococcal Herpes zoster	Number of vaccine recipients	6 months	≥50	122/249	Serious risk
Cebollero et al., 2020	United States	Non-RCT^a^	Community^d^	Advocator^f,g,h,i,m^ /Immuniser	Monitoring system^l^	Pharmacists ensure stock availability, promoting,screening, vaccine administration,education, creation of vaccine workflow for HCP with daily and weekly support visits. Comparison: Usual care	Human papillomavirus	Number of vaccine doses administered	2 months	18–26	80/241	Serious risk
Deslandes et al., 2020	United Kingdom	Non-RCT^b^	Community^c^	Immuniser	Monitoring system^l^	Pharmacists invlovement in NHS Seasonal Flu Vaccination programe as immuniser. Comparison: Vaccination administered by CPs during 6 flu seasons and vaccination administered in other facilites (GP, etc.)	Influenza	Number of vaccine recipients	6 years	≥18	635,957/552,966	Critical risk
Fathima et al., 2021	Australia	Non-RCT^a^	Community^c^	Advocator^f,i^	Monitoring system^l^	Consultant pharmacists provided referrals to GP for patients who had overdue vaccinations for influenza and pneumonia at visit 1 (baseline) and prompt the patient for immunisation at visti 2 and 3. Comparison: Usual care	Influenza Pneumococcal	Number of vaccine recipients	6 months	40–80	27/37	Serious risk
Gatwood et al., 2022	United States	Non-RCT^a^	Community^c^	Advocator^i^/Immuniser	Monitoring system^j^	A record system (code) able to detect first dose and provide alert for second dose adminisration (at least 8 weeks post 1st dose). The alert will be notified untill 2nd dose is given or after 6 months. The alert prompt pharmacist to call patients for the second dose. Comparison: Usual care	Herpes zoster	Number of vaccine doses administered	8 months	≥50	71,459/41,982	Moderate risk
Goldsworthy et al., 2022	United States	Non-RCT^a^	Community^d^	Advocator^f,i^	Monitoring system^l^	Pharmacists provided telephone outreach programe in assesing vaccination assesment, answer frequently asked questions, address common concern and facilitate vaccine administration. Comparison: Usual care	Pneumococcal Influenza	Number of vaccine recipients	2 months	≥19	398/1591	Low risk
Goode et al., 2023	United States	Non-RCT^a^	Community^d^	Advocator^f,g,h^	Monitoring system^l^	A lead champion (pharmacist) to oversee the programe. Healthcare team (includes pharmacist) were responsible for promoting influenza vaccines, providing staff education and supporting new clinic procedures Comparison:Usual care	Influenza	Number of vaccine recipients	4 months	≥18	2623/2885	Moderate risk
Heaton et al., 2022	United States	RCT	Community^c^	Advocator^i^/Immuniser	Monitoring system^j^	A technology system that enable pharmacists to query on patients immunisation records from any states immunisation information system to identify vaccine recommendations. Comparison: No intervention	Influenza Pneumococcal Herpes zoster Td/Tdap	Number of vaccine doses administered	3 months	≥19	16,841/17,062	Some concerns
Howe et al., 2022	New Zealand	Non-RCT^a^	Community^c^	Advocator^i^/Immuniser	Monitoring system^n^	Funding and promoting pertussis vaccines in pregnancy in pharmacies in between 3 regions (intervention). Comparison: Usual care	Tdap	Number of vaccine doses administered	3 years	12–50	11,748/3581	Low risk
Klassing et al., 2018	United States	RCT	Community^c^	Advocator^f,i^	Monitoring system^l^	Particpants randomised to 3 arms phone call, mailed, and no intervention. Both phone call and letter referenced the 2014 CDC immunisation schedule and guidelines. Comparison: No intervention	Influenza Pneumococcal	Number of vaccine recipients	1 month	≥18	280/140	Some concerns
Ozdemir et al., 2023	Turkey	RCT	Institution	Advocator^f,i^	Monitoring system^l^	Pharmacist provide face to face education on vaccine. Comparison: Usual care	Pneumococcal	Number of vaccine recipients	3 months	≥18	114/107	High risk
Page et al., 2020	United States	Non-RCT^b^	Community^c^	Advocator^i^/Immuniser	Monitoring system^l^	Pharmacist identify patients diagnosed with diabetes and is without history of vaccination. Pharmacists educated and recommended that the patient receive the vaccine. Comparison: Usual care	Pneumococcal	Number of vaccine recipients	4 months	≥19	68/351	Serious risk
Rihtarchik et al., 2018	United States	Non-RCT^a^	Hospital	Advocator^,^^i^	Monitoring system^j^	Pharmacy driven electronic tracking system that allows pharmacists to document and use tracking tools within the EMR to ensure appropriate vaccinations were administered to asplenic patient. Comparison: Usual care	Pneumococcal Meningococcal Haemophilus influenza type B	Number of vaccine doses administered	2 years	≥18	119/242	Moderate risk
Scherrer et al., 2020	United States	Non-RCT^a^	Hospital	Advocator^f,i^	Monitoring system^l^	Pharmacists provide post-splenectomy counselling that describes the recommended vaccinations and their importance in disease prevention.Patients are provided with handouts contains information on vaccines. Comparison: Usual care	Pneumococcal Meningococcal ACWY Meningococcal serogroup B	Number of vaccine doses administered	6 months	≥18	31/95	Moderate risk
Stolpe & Choudhry, 2019	United States	RCT	Community^c^	Immuniser	Monitoring system^j^	A vaccination prompt system was used to inform patients, that they are scheduled to receive and offered either pneumococcal vaccine, herpes zoster vaccine, or both. If patient accepted a notification will be relayed to the pharmacists. Comparison: No intervention	Pneumococcal Herpes zoster	Number of vaccine doses administered	10 months	≥65	11,009/10,962	Some concerns
Villaverde Piñeiro et al., 2022	Spain	Non-RCT^b^	Hospital	Advocator^f,i^	Monitoring system^l^	Cohort A (hospital A& intervention) received phone call from pharmacists and nurses on importance of influenza vaccination, gave campaign dates, and directions on how to arrange an appointment for the vaccine to be administered. Comparions: Cohort B (hospital B) no intervention	Influenza	Number of vaccine recipients	1 month	≥65	148/207	Moderate risk

RCT, randomised controlled/clinical trial; dTap, tetanus, diphtheria, pertussis; Td, tetanus, diphtheria.

Non-RCT: ^a^Before & after, ^b^Cohort/longitudinal with minimum two groups.

Community: ^c^Comunity pharmacy, ^d^Primary healthcare clinics, ^e^Community centre.

Advocator: ^f^Education, ^g^Training, ^h^Campaign, ^i^Recruitment, ^m^Stocking vaccine.

Monitoring system: ^j^Technology based, ^k^State law regulations, ^l^Quality improvement programme, ^n^Funding.

Eleven of the non-RCTs were before-and-after studies. Three of them (Bacci et al., 2019; Gatwood et al., 2022; Rihtarchik et al., 2018) evaluated the impact of technology-based systems that supported strategies by pharmacists to increase vaccination uptake. One study (Howe et al., 2022) assessed the impact of providing funding to community pharmacies to encourage vaccination uptake. Seven studies (Abu-rish et al., 2021; Bayraktar-Ekincioglu et al., 2022; Cebollero et al., 2020; Fathima et al., 2021; Goldsworthy et al., 2022; Goode et al., 2023; Scherrer et al., 2020) evaluated the implementation of a quality improvement programme by pharmacists to improve immunisation rates. In these studies, a comparison was made of 20 different comparator interventions against pharmacist-led interventions. Pharmacist-led approaches included pharmacists reviewing vaccination history and recommending vaccines to potential recipients or to the doctors in-charge (recruitment) (8 studies, 40%) (Abu-rish et al., 2021; Bayraktar-Ekincioglu et al., 2022; Cebollero et al., 2020; Fathima et al., 2021; Goldsworthy et al., 2022; Goode et al., 2023; Rihtarchik et al., 2018; Scherrer et al., 2020), patient education (7 studies, 35%) (Abu-rish et al., 2021; Bayraktar-Ekincioglu et al., 2022; Cebollero et al., 2020; Fathima et al., 2021; Goldsworthy et al., 2022; Goode et al., 2023; Scherrer et al., 2020), training (2 studies, 10%) (Cebollero et al., 2020; Goode et al., 2023), organising campaigns (2 studies, 10%) (Cebollero et al., 2020; Goode et al., 2023), and stocking up on vaccines (1 study, 5%) (Cebollero et al., 2020). Most of these studies were from the USA (7 studies, 63.6%) (Bacci et al., 2019; Cebollero et al., 2020; Gatwood et al., 2022; Goldsworthy et al., 2022; Goode et al., 2023; Rihtarchik et al., 2018; Scherrer et al., 2020) and one study each from Turkey (9.1%) (Bayraktar-Ekincioglu et al., 2022), New Zealand (9.1%) (Howe et al., 2022), Jordan (9.1%) (Abu-rish et al., 2021) and Australia (9.1%) (Fathima et al., 2021). These studies were primarily conducted in community settings: four community pharmacies (36.3%) (Bacci et al., 2019; Fathima et al., 2021; Gatwood et al., 2022; Howe et al., 2022), three primary healthcare clinics (27.2%) (Cebollero et al., 2020; Goldsworthy et al., 2022; Goode et al., 2023), one community centre (9.1%) (Goldsworthy et al., 2022), and three hospitals (27.2%) (Abu-rish et al., 2021; Rihtarchik et al., 2018; Scherrer et al., 2020).

The remaining four non-RCTs (Are et al., 2022; Deslandes et al., 2020; Page et al., 2020; Villaverde Piñeiro et al., 2022) were categorised as cohort/longitudinal studies with a minimum of two groups. Three of these studies (Deslandes et al., 2020; Page et al., 2020; Villaverde Piñeiro et al., 2022) evaluated the impact of quality improvement programmes conducted by pharmacists to increase immunisation rates while the other study (Are et al., 2022) investigated the impact of having given pharmacists the authority to immunise on immunisation rates in several different states. Several studies evaluated the position of pharmacists as immunisers (3 studies, 50%) (Are et al., 2022; Deslandes et al., 2020; Page et al., 2020), reviewing vaccination history and recommending vaccines to potential vaccine recipients or to the doctors in-charge (recruitment –2 studies, 33.3%) (Page et al., 2020; Villaverde Piñeiro et al., 2022), and patient education (1 study, 16.7%) (Villaverde Piñeiro et al., 2022). Two of those studies were from the USA (50%) (Are et al., 2022; Page et al., 2020), and one study each from Spain (25%) (Villaverde Piñeiro et al., 2022), and the UK (25%) (Deslandes et al., 2020). Community pharmacies were the main settings in those three studies (75%) (Are et al., 2022; Deslandes et al., 2020; Page et al., 2020) followed by hospital (25%) (Villaverde Piñeiro et al., 2022).

### Quality of evidence

A total of 19 studies consisting of both RCTs and non-RCTs, with diverse interventions were successfully assessed for risk of biases. The CASP checklist evaluated the methodological soundness of the studies. Among the non-RCTs, two studies (13.3%) were classified as of high quality, ten studies (66.7%) as of moderate quality, and three studies (20%) as of low quality. For RCTs, two articles (50%) were of high quality, and two articles (50%) were determined to have been of poor quality.

Using the Cochrane ROB 2 tool, we observed varying levels of risk of bias among the 15 non-RCTs. One study (6.7%) had a low risk of bias, eight studies (53.3%) had a moderate risk, four studies (26.7%) had a serious risk, and two studies (13.3%) had a critical risk. For the RCT category, none of the studies was deemed to have a low risk of bias. All the studies had unclear risks for selective reporting bias. One study (Ozdemir et al., 2023) had an overall high risk of bias, while three studies had some concerns regarding their risks of bias (Heaton et al., 2022; Klassing et al., 2018; Stolpe & Choudhry, 2019).

In terms of randomisation, there were some concerns. Several studies lacked information on allocation concealment (Heaton et al., 2022; Klassing et al., 2018; Ozdemir et al., 2023; Stolpe & Choudhry, 2019), while two studies employed stratified randomisation (Heaton et al., 2022; Stolpe & Choudhry, 2019) and others used simple randomisation (Klassing et al., 2018; Ozdemir et al., 2023). The nature of the intervention made blinding of participants and pharmacists impractical, but this lack of blinding did not significantly impact the deviations from intended interventions in those four studies.

While there were instances of loss of follow-up in all four studies, the number of participants with missing outcome data was small, leading to a low risk of bias in their assessment. Three studies had a low risk of bias in outcome measurement, as the assessment was unlikely to be influenced by knowledge of the intervention received. However, one study demonstrated a high risk of bias in the outcome measurement due to the potential influence of pre-existing knowledge of the intervention.

We observed that the two studies by Fathima et al. (2021) and Cebollero et al. (2020) received a lower number of ‘yes' responses for the validity questions of the CASP checklist (Supplement II). This finding is further supported by the ROB 2 assessment, where these two studies were classified as having a serious risk of bias.

**Figure 2.**
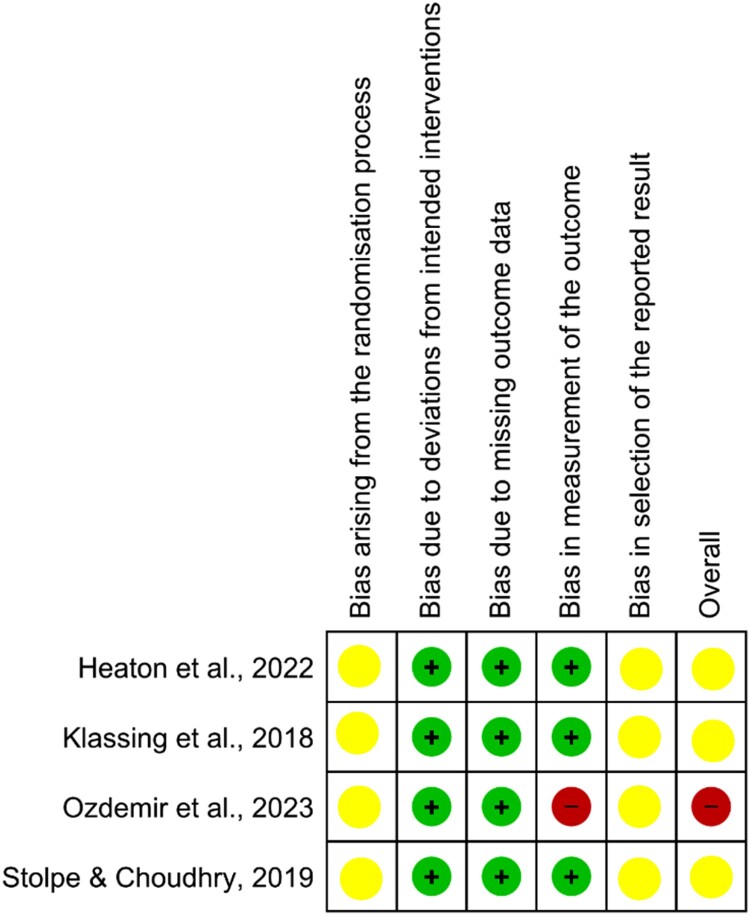
A detailed description of the ROB assessment for RCTs.

**Figure 3.**
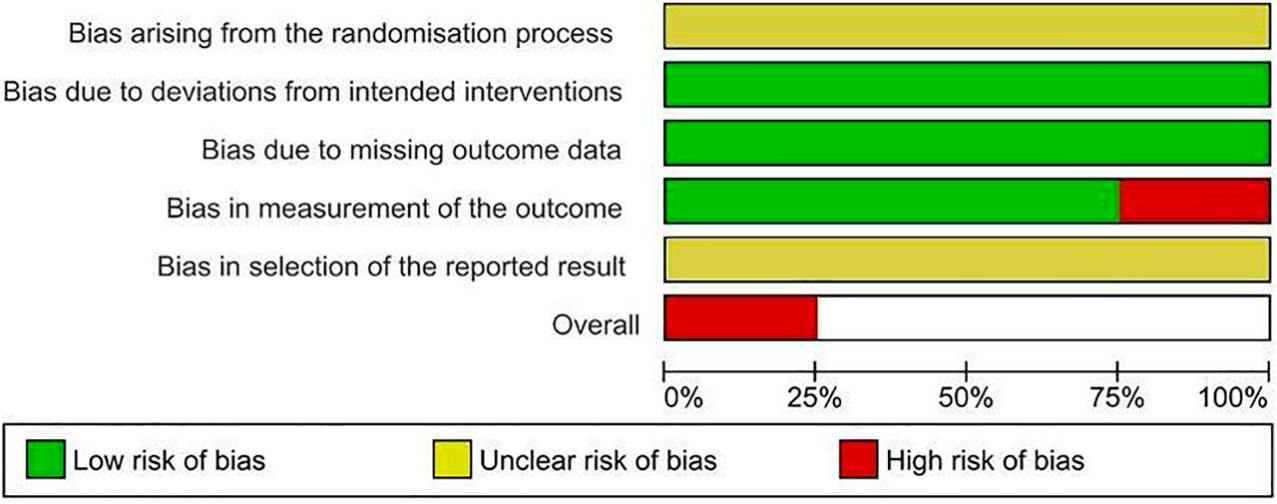
Summary plot of ROB assessment for RCTs.

**Figure 4.**
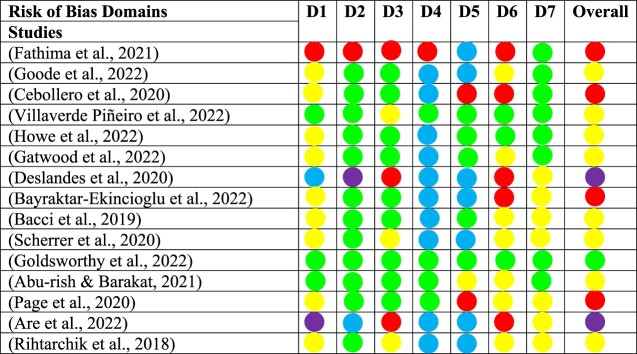
A detailed description of the ROB assessment for non-RCTs.

**Figure 5.**
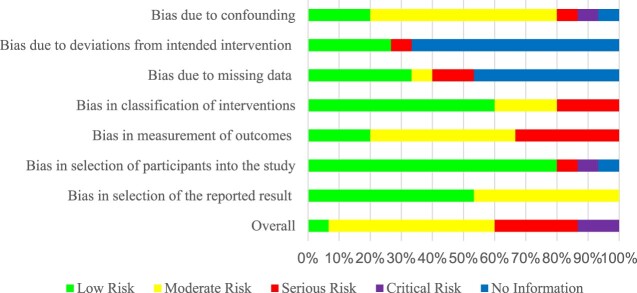
Summary plot of ROB assessment for non-RCTs.

A detailed description of the ROB assessment for RCTs are displayed in Figures 2 and 3. The quality of evidence for non-RCTs are described in Figures 4 and 5. These assessments provided important insights into the strengths and weaknesses of the included studies, ensuring that we interpret and consider their findings in consideration of the risk of bias and methodological quality.

### Effect sizes

There are 19 studies included for data analysis and the risk ratio for each study is summarised in Table 2.

**Table 2.** Summary of analyses of the impact of pharmacist interventions on immunisation uptake.

Study (author, year)	Risk ratio (95% confidence interval), inverse variance, random effect
Abu-rish et al., 2021	1.03 [0.86, 1.23]
Are et al., 2022	0.98 [0.97, 0.99]
Bacci et al., 2019	1.20 [1.19, 1.21]
Bayraktar-Ekincioglu et al., 2022	0.18 [0.09, 0.37]
Cebollero et al., 2020	1.44 [1.05, 1.99]
Deslandes et al., 2020	20.10 [19.11, 21.13]
Fathima et al., 2021	1.73 [1.23, 2.43]
Gatwood et al., 2022	1.05 [1.04, 1.05]
Goldsworthy et al., 2022	1.99 [1.76, 2.25]
Goode et al., 2023	1.56 [1.46, 1.67]
Heaton et al., 2022	1.19 [1.11, 1.27]
Howe et al., 2022	1.01 [0.99, 1.03]
Klassing et al., 2018	0.68 [0.52, 0.89]
Ozdemir et al., 2023	3.29 [1.47, 7.35]
Page et al., 2020	1.11 [0.79, 1.55]
Rihtarchik et al., 2018	1.35 [0.94, 1.96]
Scherrer et al., 2020	5.04 [2.14, 11.87]
Stolpe & Choudhry, 2019	1.04 [0.87, 1.25]
Villaverde Piñeiro et al., 2022	5.31 [2.74, 10.32]
Pooled RR: 1.51 [1.28, 1.77] Heterogeneity: τ^2^ = 0.10; *χ*^2^ = 14230.16, df = 18 (*P* < 0.00001); *I*² = 100% Test for overall effect: *Z* = 4.99 (*P* < 0.00001)	

### Meta-analyses

#### Pooled risk ratios (RRs)

**Figure 6.**
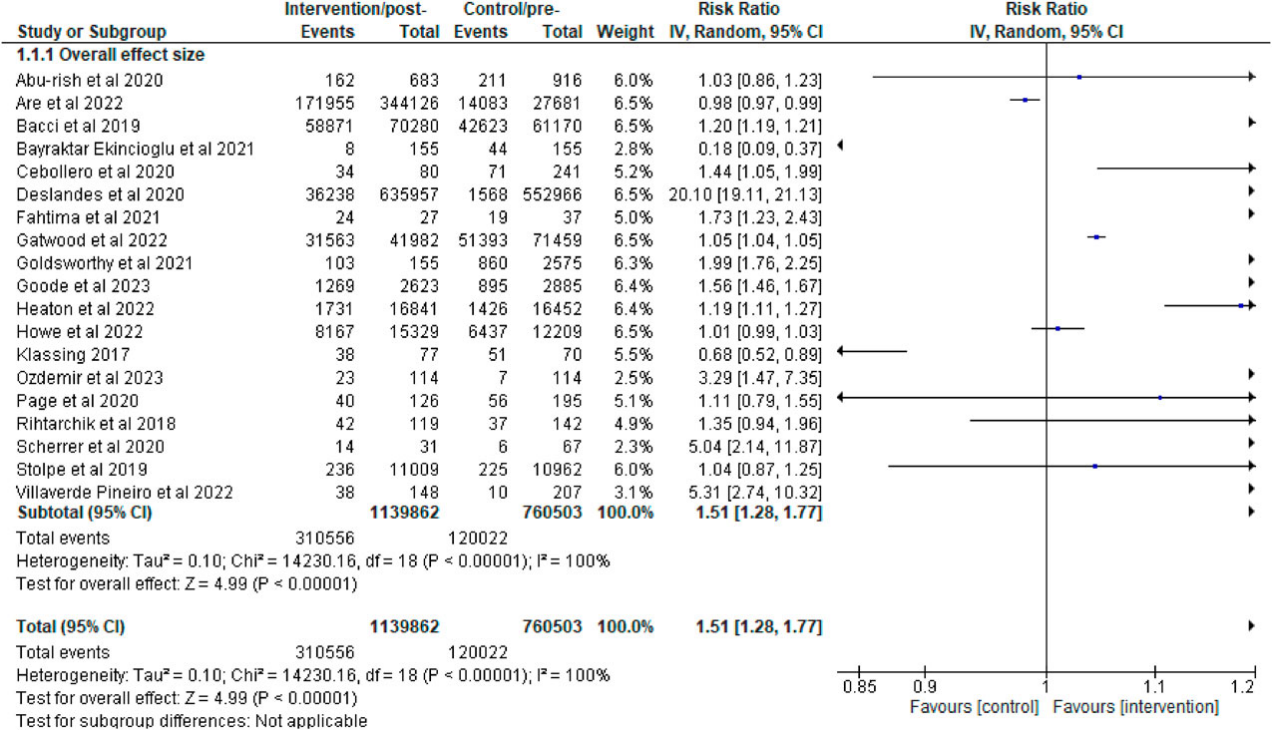
Overall impact of pharmacist’s involvement on immunisation uptake.

A meta-analysis was performed which resulted in an estimated pooled RR of 1.51 (1.28, 1.77) and that indicated a significant positive impact of pharmacist interventions on immunisation uptake. However, there is a significant heterogeneity in the outcome measures of the studies (*I*^2^ = 100%, *p* < 0.05) that require cautious interpretation of its results (Figure 6).

#### Subgroup analyses

**Figure 7.**
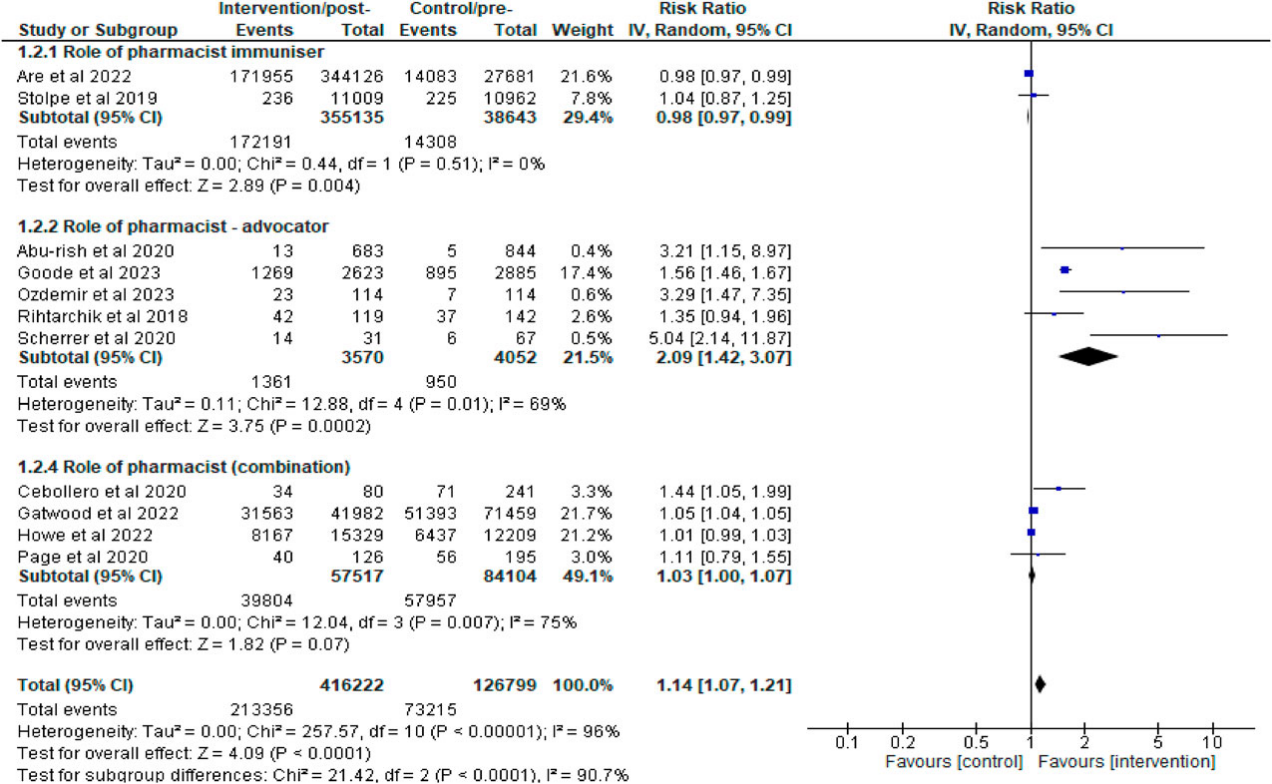
Subgroup analysis on the impact of pharmacist intervention on immunisation uptake according to their roles as immunisers, advocators, or both.

To address the significant heterogeneity between studies, subgroup analyses were performed by firstly categorising the studies according to the role of pharmacists (Figure 7). The findings revealed that, when pharmacists acted as advocators, through education, training, campaign, recruitment, inventory control or stocking up vaccines, a significant positive impact with a lesser extent of heterogeneity, was observed on the immunisation uptake [RR 2.09 (1.42,3.07; *I*^2^ = 69%)]. The analysis involved five (5) studies (Abu-rish & Barakat, 2021; Goode et al., 2023; Ozdemir et al., 2023; Rihtarchik et al., 2018; Scherrer et al., 2020).

Firstly, an analysis of four studies (Cebollero et al., 2020; Gatwood et al., 2022; Howe et al., 2022; Page et al., 2020) where pharmacists acted as both immunisers and advocators, revealed significantly improved immunisation uptake with a lower degree of heterogeneity [RR 1.03 (1.00, 1.21; *I*^2^ = 75%)]. Nevertheless, an analysis of studies where pharmacists acted as immunisers alone did not result in an overall improvement in the immunisation uptake.

**Figure 8.**
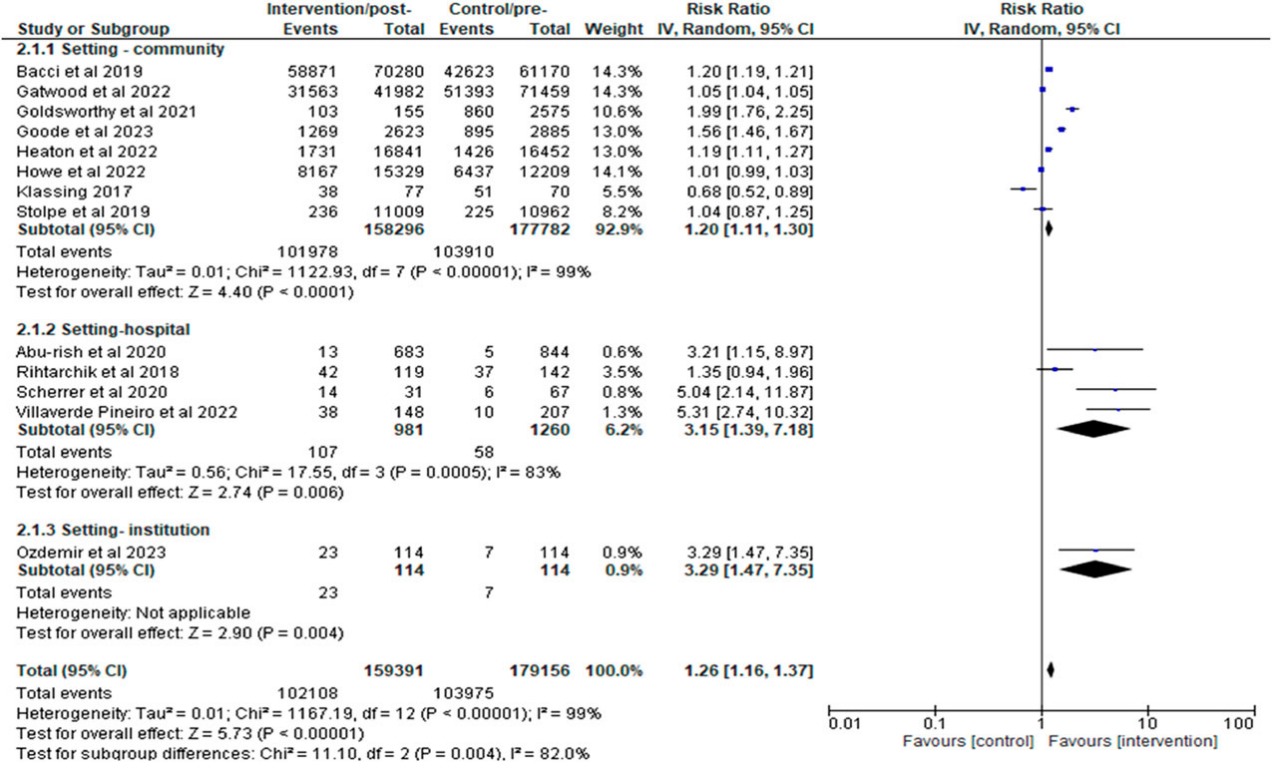
Subgroup analysis of the impact of pharmacist interventions on immunisation uptake according to setting (hospital, community, or institution).

Secondly, a subgroup analysis of the study settings demonstrated a significant association between increased immunisation uptake and pharmacist involvement in two settings [community: RR 1.20 [1.11, 1.30] and hospital: RR 3.15 [1.39, 7.18], (both with *p* values < 0.05)]. This is presented in Figure 8. A pooled effect was not computed for ‘institution' as only one study was conducted in this setting. All the analyses were associated with a high heterogeneity (*I*^2^ > 80%).

### Publication bias

**Figure 9.**
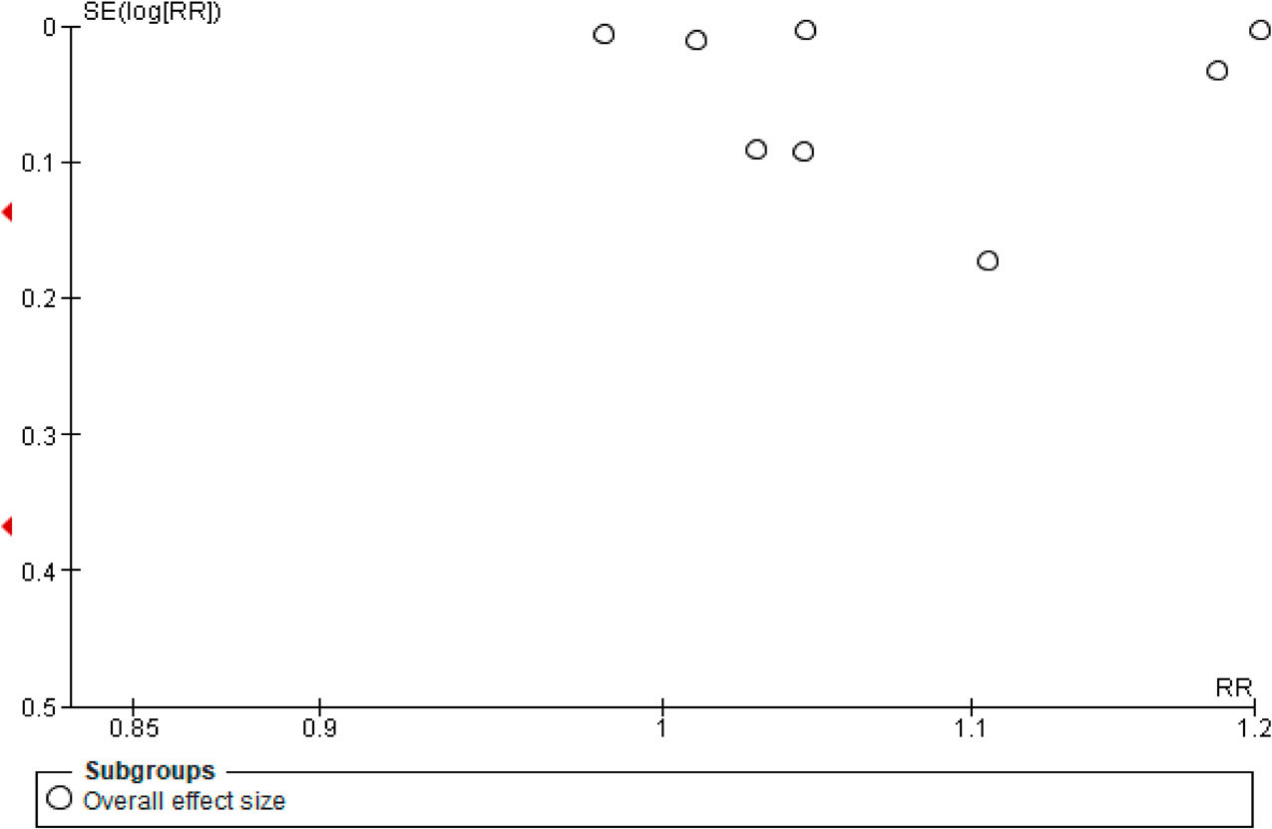
Funnel plot demonstrating the presence of publication bias (overall effect size).

**Figure 10.**
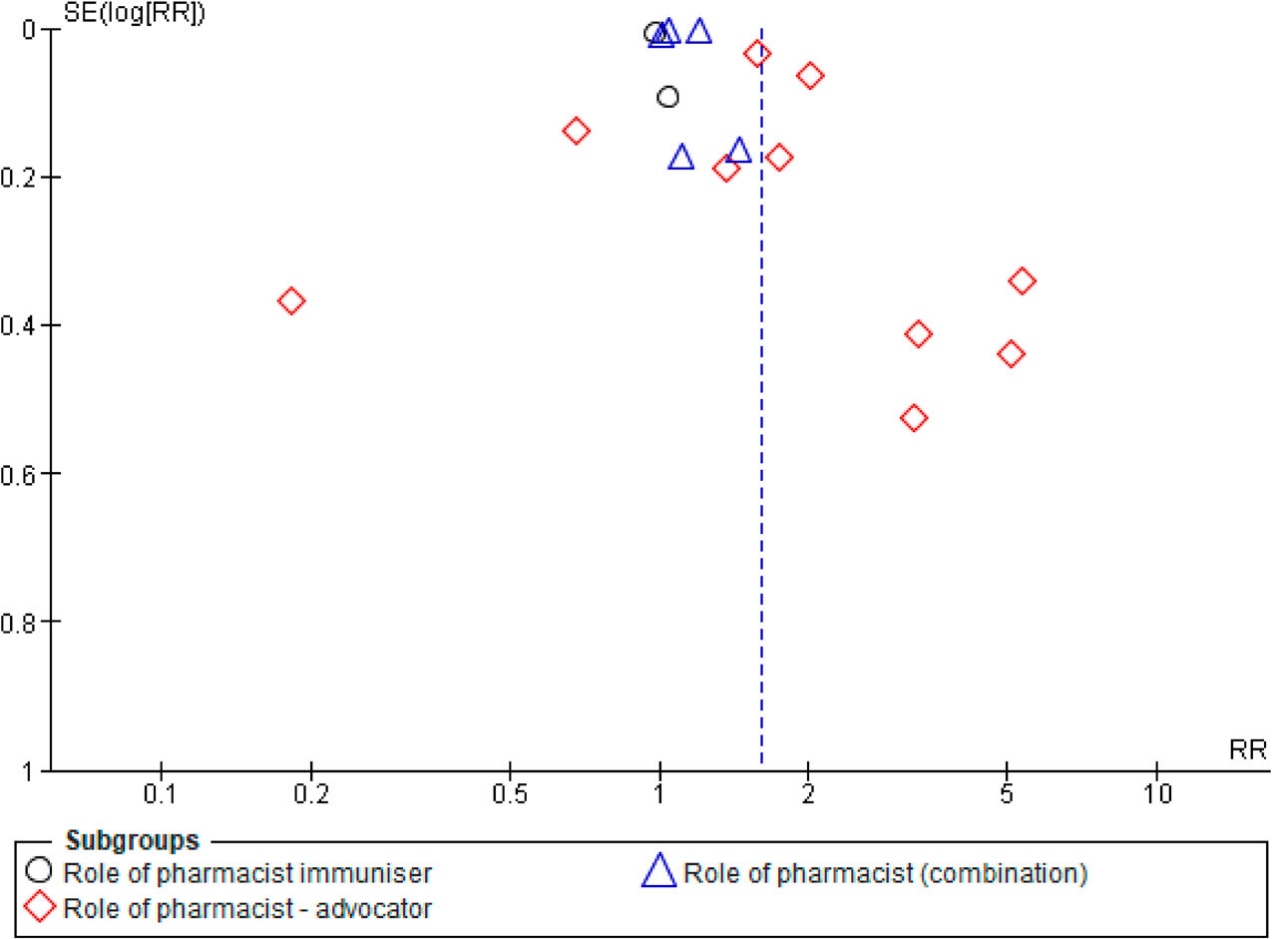
Funnel plot demonstrating the presence of publication bias (subgroup analysis of pharmacist roles).

**Figure 11.**
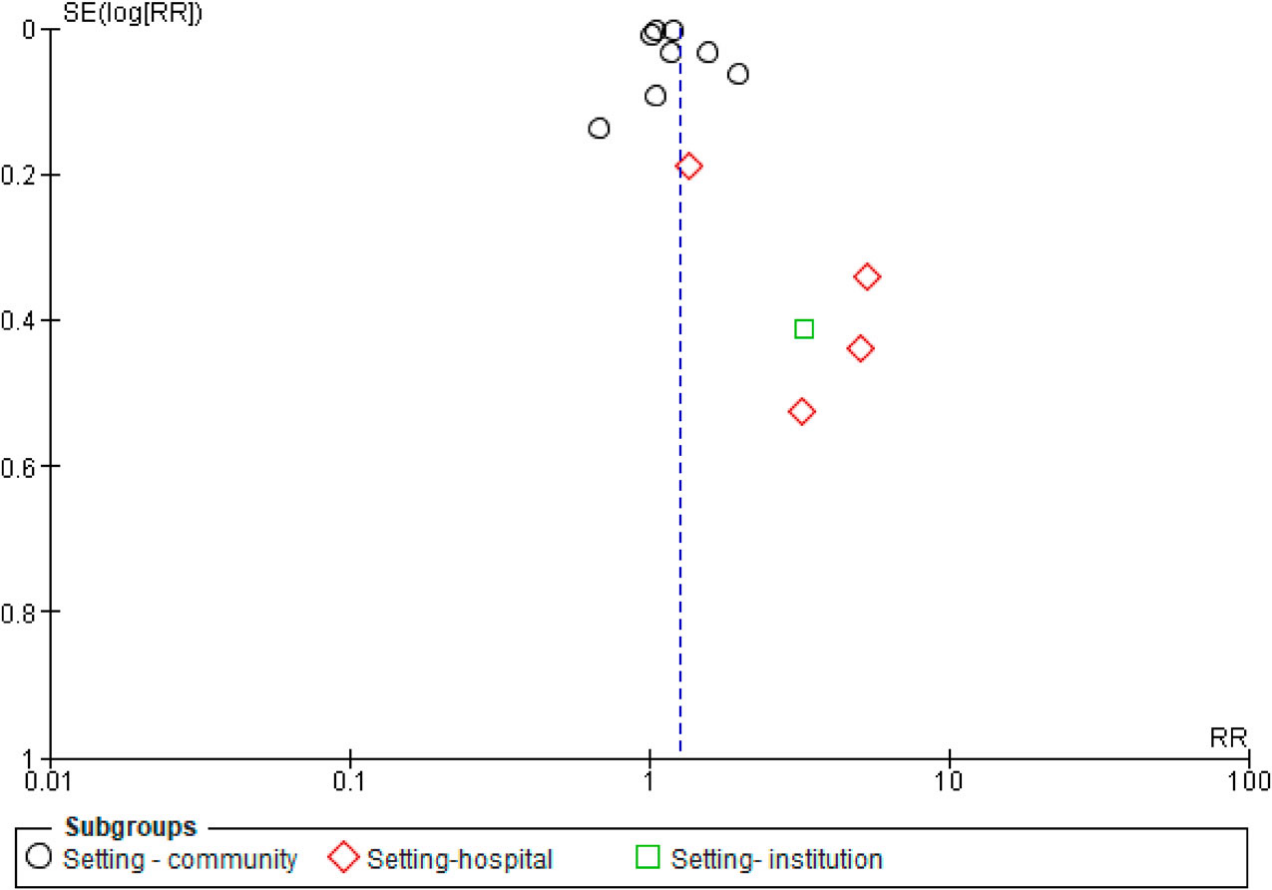
Funnel plot demonstrating the presence of publication bias (subgroup analysis of study setting).

Assessments of publication biases are illustrated through funnel plots, all of which had shown asymmetrical outputs for overall effect sizes (Figure 9), and for the subgroup analyses [(Figure 10 – role of pharmacists) (Figure 11 – study setting)], thus indicating the presence of publication bias.

## Discussion

Several studies had demonstrated the favourable impact of interventions by pharmacists on public immunisation. Findings from both non-RCTs and RCTs indicated that in comparison to usual care, pharmacists, regardless of their responsibilities as facilitators, advocators, or immunisers, elicited a substantial impact on immunisation rate. They contributed to higher vaccination uptake by up to 51%, as compared to usual care or when pharmacists were not part of the programme.

In this meta-analysis, the pooled effect size of immunisation uptake was significantly increased when pharmacists acted as advocators (participated in education, training, campaigns, recruitment, inventory control/stocking vaccine). This finding was in line with a meta-analysis by Le et al. (2022) that demonstrated a 31% increase in vaccination uptake as a consequence of programmes conducted in which pharmacists acted as advocators (RR 1.31 [1.17, 1.48]) (Le et al., 2022). Another systematic review and meta-analysis had demonstrated that following the contribution of pharmacists as educators (in our study, they were categorised as advocators), a positive increase in immunisation rates as compared to usual care (RR 2.96 [1.02, 8.59]) (Isenor et al., 2016) was observed.

With the participation of pharmacist immunisers or administrators, Le et al. (2022) and Isenor et al. (2016) both found significant positive impact on immunisation rates (RR 1.14 [1.12–1.15] and RR 2.64 [1.81, 3.85] respectively). However, our study found that when pharmacists acted as immunisers alone, they did not significantly improve vaccination rates when compared to usual care.

A systematic review and meta-analysis by Lan et al. (2022) concluded that pharmacists acting as immunisers, advocators, or both had beneficial effects on immunisation uptake, particularly for influenza vaccines in the USA and several high-income countries (Lan et al., 2022). In a previously published meta-analysis by Murray et al. (2021), the authors reported an increase in vaccination rate by 24% (RR 1.24 [1.05, 1.47]) (Murray et al., 2021). Baroy et al. (2016) also revealed a significant positive impact of pharmacist involvement on immunisation rates as compared to usual care. The reported RR was 2.95 (2.25, 3.87) (Baroy et al., 2016). Similar to our study, a high heterogeneity between studies was reported in both the studies (Baroy et al., 2016; Murray et al., 2021).

Evidence from RCTs demonstrated that pharmacist participation in vaccination activities in both community and hospital settings had a positive impact on immunisation rates, particularly for influenza vaccine in the community settings. We performed a series of sensitivity analyses for all the studies and observed that our results were robust. The sensitivity analyses indicated that pharmacists who served in the hospital or community offered greater contribution to the public's immunisation uptake. Le et al. (2022) also reported similar significant positive results [RR 1.17 (1.06, 1.28)] for both community and [RR 2.82 (1.13, 7.03)] hospital settings (Le et al., 2022).

### Recommendations for policy and practice

Our findings further support the notion that pharmacists make valuable team members of vaccination providers. The provision of services by pharmacists and their engagement in immunisation programmes either as immunisers, advocators, or in combination roles are crucial to corroborate the professional role played. The effectiveness of community pharmacist-led vaccination services that led to increasing vaccination and vaccine delivery rates, and in improving patient satisfaction was successfully demonstrated (Youssef et al., 2021). To further realise national and international immunisation goals, and before pharmacist immunisers are authorised to administer vaccines, they are required to complete immunisation training programmes and obtain valid certification in cardiopulmonary resuscitation and first aid (Houle et al., 2019). In Malaysia, for instance, during the COVID-19 pandemic, pharmacists were given an opportunity to be part of the mass immunisation program as immunisers under the National COVID-19 Immunisation Program (PICK). To ensure that the pharmacists were adequately trained to provide vaccination, a national university, Universiti Sains Malaysia (USM), in collaboration with the Malaysia Pharmacists Society (MPS) had introduced Certified Training Programme on Immunisation for Pharmacists (CTPIP) (Sharmika et al., 2022).

Additionally, it is essential to better understand barriers and challenges in implementing immunisation programmes. This knowledge can then enable the development of more tailored and efficient pharmacist interventions that leads to improved immunisation rates. Interestingly, low vaccine confidence and government mistrust were among the top reasons cited when the public opted out of vaccination programmes (Shah & Coiado, 2023). The implementation of a less successful government programme likely contributed to the discrepancy in anticipated vaccine hesitancy and actual vaccination rates (Jafar et al., 2022; Zin et al., 2022).

Future research could examine patients’ perceived need for immunisation in tandem with advocating patient-centred health outcomes.

### Strengths and limitations

While the presence of risks of biases was inevitable, the inclusion of RCTs and non-RCTs increased the likelihood of retrieving high quality studies. This was an added advantage when compared to previous meta-analyses of similar nature that had been performed.

In comparison to the number of RCTs, the greater number of non-RCTs (before and after, cohort or longitudinal studies with minimum two groups) that was retrieved contributed to the study's inherent risk of bias. Nevertheless, the non-RCTs reflected the real-world scenario, where it was often times impossible to blind or randomise patients on a day-to-day practical basis when providing pharmaceutical care.

In addition, our data analyses did reveal high levels of heterogeneity (> 80%). It was challenging to conclude or determine the exact mechanism or approach which contributed significantly to the overall increased immunisation rates. Nevertheless, subgroup analyses were performed, several of which revealed heterogeneity but then to a lesser extent. Previous researchers had also alluded to the challenges faced in handling heterogenous data (Baroy et al., 2016; Isenor et al., 2016; Le et al., 2022; Murray et al., 2021).

Finally, the findings of the meta-analyses should be interpreted appropriately in consideration of regional jurisdiction and local healthcare settings.

## Conclusion

While the evidence for pharmacist immunisers were mixed, their contribution to immunisation programmes boosted public vaccination rate. Pharmacists demonstrated leadership and acquired indispensable advocator roles in the community and hospital settings. Irrespective of the responsibilities undertaken, pharmacist interventions contributed up to 51% increment in public vaccination rate, in comparison to control groups which received usual care. Future research could explore the depth of engagement and hence the extent of influence on immunisation uptake.

## Supplementary Material

Supplemental Material

## Data Availability

The data that support the findings of this study are available in the supplementary material section of this article.
