# Supplementary material for: Impact of pharmacist interventions on immunisation uptake: a systematic review and meta-analysis
Source: J Pharm Policy Pract. 2023 Dec 7;17(1):2285955. doi: 10.1080/20523211.2023.2285955 (PMC10775721; doi:10.1080/20523211.2023.2285955)
Supplement: Supplemental Material [file JPPP_A_2285955_SM0462.docx]

# Supplementary Materials

## Supplement 1: Search Strategies

### Full Search Strategy – Keyword

| **“THE IMPACT OF PHARMACIST INVOLVEMENT ON IMMUNIZATION UPTAKE AND OTHER OUTCOMES: A SYSTEMATIC REVIEW AND META-ANALYSIS”** | | | | |
| --- | --- | --- | --- | --- |
| ***QUESTION*** | What is the impact of pharmacist involvement on immunisation uptake and other outcomes? | | | |
| ***OBJECTIVE*** | To determine the impact of pharmacist involvement on immunization uptake and other outcomes | | | |
| ***POPULATION*** | | ***INTERVENTION*** | ***COMPARISON*** | ***OUTCOME*** |
| Individual received immunization. | | Pharmacist involvement on immunization. | Pre-receiving pharmacy care.  Non-receiving pharmacy care.  Usual care. | Immunization outcomes. |
| **KEYWORD** | | | | |
| *Considering outcome among all population.*  *Not to be included in search strategy.* | | Pharmacist,  Pharmacy,  Dispenser,  Apothecary,  Druggist,  Drug store,  Chemist,  Pharmaceutical care. | *Search for All Possible Outcome.*  *(To be filtered out based on inclusion & exclusion criteria).* | Immunization,  Vaccination,  Booster,  Inoculation. |

### Search Filter: Search Terms

| ***BLOCK 1*** | ***BLOCK 2*** | **BLOCK 1 + BLOCK 2** |
| --- | --- | --- |
| ***SEARCH TERMS*** | | |
| Pharmacy, pharmacist, pharmacies, pharmaceutical, pharmacologist. | Immunization, immunisation, immune, immunity. | (BLOCK 1) AND (BLOCK 2) |
| Dispensaries, dispenser, dispensary. | Vaccination, vaccine, vaccinate, vaccinated, vaccinating. |  |
| Apothecary, apothecaries. | Booster, boost, boosted. |  |
| Druggist, drugstore, drug store. | Inoculation, inoculate, inoculating, inoculated. |  |
| Chemist |  |  |
| Pharmaceutical care, pharmacy care, pharmacies care, pharmacist care. |  |  |

### Search Filter: Search String

| ***SEARCH FILTER*** | | |
| --- | --- | --- |
| **SCOPUS** | | |
| **Search** | **Query** | |
| #1 | TITLE-ABS-KEY((pharmacy OR pharmacist OR pharmacies OR pharmaceutical OR pharmacologist OR dispensaries OR dispenser OR dispensary OR Apothecary OR apothecaries OR Druggist OR drugstore OR Chemist) OR (“drug store” OR “pharmaceutical care OR “pharmacy care” OR “pharmacies Care” OR “pharmacist care”) | |
|  | Date of search | 22/3/2023 |
|  | No of article (result) | 25,074 |
| #2 | TITLE-ABS-KEY(Immunization OR immunisation OR immune OR immunity OR Vaccination OR vaccine OR vaccinate OR vaccinated OR vaccinating OR booster OR boost OR boosted OR inoculate OR inoculation OR inoculating OR inoculated) | |
|  | Date of search | 22/3/2023 |
|  | No of article (result) | 5,766,567 |
| #1 AND #2 | TITLE-ABS-KEY((pharmacy OR pharmacist OR pharmacies OR pharmaceutical OR pharmacologist OR dispensaries OR dispenser OR dispensary OR Apothecary OR apothecaries OR Druggist OR drugstore OR Chemist) OR (“drug store” OR “pharmaceutical care OR “pharmacy care” OR “pharmacies Care” OR “pharmacist care”) AND TITLE-ABS-KEY(Immunization OR immunisation OR immune OR immunity OR Vaccination OR vaccine OR vaccinate OR vaccinated OR vaccinating OR booster OR boost OR boosted OR inoculate OR inoculation OR inoculating OR inoculated) | |
|  | Date of research | 22/3/2023 |
|  | No of article (result) | 3,697 |
| #3 | Filter date: 2019 – Februari 2023 | 736 |
| #4 | Filter language: English | 730 |
| **WOS** | | |
| #1 | TS=((pharmacy OR pharmacist OR pharmacies OR pharmaceutical OR pharmacologist OR dispensaries OR dispenser OR dispensary OR Apothecary OR apothecaries OR Druggist OR drugstore OR Chemist) OR (“drug store” OR “pharmaceutical care OR “pharmacy care” OR “pharmacies Care” OR “pharmacist care”) | |
|  | Date of research | 27/3/2023 |
|  | No of article (result) | 83,033 |
| #2 | TS=(Immunization OR immunisation OR immune OR immunity OR Vaccination OR vaccine OR vaccinate OR vaccinated OR vaccinating OR booster OR boost OR boosted OR inoculate OR inoculation OR inoculating OR inoculated) | |
|  | Date of research | 27/3/2023 |
|  | No of article (result) | 392,341 |
| #1 AND #2 | TS=((pharmacy OR pharmacist OR pharmacies OR pharmaceutical OR pharmacologist OR dispensaries OR dispenser OR dispensary OR Apothecary OR apothecaries OR Druggist OR drugstore OR Chemist) OR (“drug store” OR “pharmaceutical care OR “pharmacy care” OR “pharmacies Care” OR “pharmacist care”) AND TS=(Immunization OR immunisation OR immune OR immunity OR Vaccination OR vaccine OR vaccinate OR vaccinated OR vaccinating OR booster OR boost OR boosted OR inoculate OR inoculation OR inoculating OR inoculated) | |
|  | Date of research | 27/3/2023 |
|  | No of article (result) | 1,214 |
| #3 | Filter date: 2019 – Feb 2023 | 615 |
| #4 | Filter language: English | 596 |
| **PUBMED** | | |
| #1 | ((((((pharmacist[MeSH Terms]) OR (pharmacist[Title/Abstract])) OR (drug store[Title/Abstract])) OR (dispenser[Title/Abstract])) OR (apothecary[Title/Abstract])) OR (druggist[Title/Abstract])) OR (chemist[Title/Abstract]) | |
|  | Date of research | 27/3/2023 |
|  | No of article (result) | 36,657 |
| #2 | (((((immunization[MeSH Terms]) OR (vaccination[MeSH Terms])) OR (immunization[Title/Abstract])) OR (vaccination[Title/Abstract])) OR (booster[Title/Abstract])) OR (inoculation[Title/Abstract]) | |
|  | Date of research | 27/3/2023 |
|  | No of article (result) | 425,612 |
| #1 AND #2 | (((((((pharmacist[MeSH Terms]) OR (pharmacist[Title/Abstract])) OR (drug store[Title/Abstract])) OR (dispenser[Title/Abstract])) OR (apothecary[Title/Abstract])) OR (druggist[Title/Abstract])) OR (chemist[Title/Abstract])) AND ((((((immunization[MeSH Terms]) OR (vaccination[MeSH Terms])) OR (immunization[Title/Abstract])) OR (vaccination[Title/Abstract])) OR (booster[Title/Abstract])) OR (inoculation[Title/Abstract])) | |
|  | Date of research | 27/3/2023 |
|  | No of article (result) | 676 |
| #3 | Filter date: 2019 – Feb 2023 | 340 |
| #4 | Filter language: English | 335 |

## Supplement II: Critical Appraisal for Included Studies

1. **The Critical Appraisal Skills Programme (CASP) Cohort score for assessing the methodological quality of the fifteen enrolled studies.**

| No | Author/Year | CASP 01 | CASP 02 | CASP 03 | CASP 04 | CASP 05 (a) | CASP 05 (b) | CASP 06 (a) | CASP 06 (b) | CASP 07 | CASP 08 | CASP 09 | CASP 10 | CASP 11 | CASP 12 |  |
| --- | --- | --- | --- | --- | --- | --- | --- | --- | --- | --- | --- | --- | --- | --- | --- | --- |
| 7 | Fathima et al (2021) | 1 | 0 | 0 | 0 | 0 | 1 | 1 | 1 | 1 | 0 | 1 | X | 1 | 1 |  |
| 12 | Goode et al (2022) | 1 | 1 | X | X | 1 | 1 | 1 | X | 1 | 0 | 1 | X | 1 | 1 |  |
| 13 | Cebollero et al (2020) | 1 | 1 | X | 1 | 0 | 0 | 0 | 0 | 1 | 0 | X | X | 1 | 1 |  |
| 27 | Villaverde Piñeiroet al (2022) | 1 | 1 | X | X | X | X | 1 | 1 | 1 | 0 | X | 1 | 1 | 1 |  |
| 31 | Howe et al (2022) | 1 | 1 | 1 | 1 | 1 | X | X | 1 | 1 | 1 | 1 | 1 | 1 | 1 |  |
| 55 | Gatwood et al (2022) | 1 | 1 | X | 0 | 1 | 1 | 1 | 1 | 1 | 1 | 1 | 1 | 1 | 1 |  |
| 64 | Deslandes et al (2020) | 1 | X | X | X | 1 | 1 | 1 | 1 | 1 | 0 | 1 | X | 1 | 1 |  |
| 83 | Bayraktar et al (2022) | 1 | X | X | X | 0 | 0 | 1 | 1 | 1 | 0 | 1 | X | 1 | 1 |  |
| 88 | Bacci et al (2019) | 1 | 1 | X | X | 0 | 0 | 1 | 1 | 1 | 0 | X | X | 1 | 1 |  |
| 107 | Scherrer et al (2020) | 1 | 1 | X | X | X | X | 1 | 1 | 1 | 0 | 1 | 1 | 1 | 1 |  |
| 108 | Claudine et al (2021) | 1 | X | X | X | X | X | 1 | 1 | 1 | X | 1 | X | 1 | 1 |  |
| 119 | Abu-rish119 et al., 2020 | 1 | 1 | 1 | 1 | X | X | 1 | 1 | 1 | 0 | 1 | 1 | 1 | 1 |  |
| 144 | Page et al (2020) | 1 | X | X | 0 | X | X | 1 | 1 | 1 | 0 | 1 | 1 | 1 | 1 |  |
| 208 | Are2 et al (2022) | 1 | X | X | X | X | X | 1 | 1 | 1 | 1 | X | X | 1 | 1 |  |
| 228 | Rihtarchik et al (2018) | 1 | X | X | X | X | X | 1 | 1 | 1 | 0 | X | 0 | 0 | 1 |  |

1. **The Critical Appraisal Skills Programme (CASP) RCT score for assessing the methodological quality of the four enrolled RCT studies.**

| No | Author/Year | | CASP 01 | CASP 02 | CASP 03 | CASP 04 | CASP 05 | CASP 06 | CASP 07 | CASP 08 | CASP 09 | CASP 10 | CASP 11 |
| --- | --- | --- | --- | --- | --- | --- | --- | --- | --- | --- | --- | --- | --- |
| 87 | Stolpe et al (2019) | | 1 | 1 | 1 | 0 | 1 | 1 | 1 | 1 | 1 | 1 | 1 |
| 95 | Klassing et al (2018) | | 1 | 1 | 1 | 0 | 1 | X | 1 | 1 | X | 1 | X |
| 111 | Heaton et al (2021) | | 1 | 1 | 1 | 0 | X | 0 | 1 | 1 | 1 | 0 | X |
| 118 | Ozdemir et al (2023) |  | 1 | 1 | 1 | 0 | 1 | 1 | 1 | 0 | 1 | 1 | 1 |

1. **CASP Analysis for non-RCT**

| **No** | **Author/Year** | **Total Yes** | **Total No** | **Total Unsure** |
| --- | --- | --- | --- | --- |
| 7 | Fathima et al (2021) | 8 | 5 | 1 |
| 12 | Goode et al (2022) | 9 | 4 | 1 |
| 13 | Cebollero et al (2020) | 6 | 5 | 3 |
| 27 | Villaverde Piñeiro et al (2022) | 8 | 1 | 5 |
| 31 | Howe et al (2022) | 12 | 0 | 2 |
| 55 | Gatwood et al (2022) | 12 | 1 | 1 |
| 64 | Deslandes et al (2020) | 9 | 1 | 4 |
| 83 | Bayraktar et al (2022) | 7 | 3 | 4 |
| 88 | Bacci et al (2019) | 7 | 3 | 4 |
| 107 | Scherrer et al (2020) | 9 | 1 | 4 |
| 108 | Claudine et al (2021) | 7 | 0 | 7 |
| 119 | Abu-rish119 et al., 2020 | 11 | 1 | 2 |
| 144 | Page et al (2020) | 8 | 2 | 4 |
| 208 | Are2 et al (2022) | 7 | 0 | 7 |
| 228 | Rihtarchik et al (2018) | 5 | 3 | 6 |

1. **CASP Analysis for RCT**

| **No** | **Author/Year** | **Total Yes** | **Total No** | **Total Unsure** |
| --- | --- | --- | --- | --- |
| 87 | Stolpe et al (2019) | 10 | 1 | 0 |
| 95 | Klassing et al (2018) | 7 | 1 | 3 |
| 111 | Heaton et al (2021) | 6 | 3 | 2 |
| 118 | Ozdemir et al (2023) | 9 | 2 | 0 |

## Supplement III: Risk of Bias Assessment

1. **Quality of Evidence from RCTs through the ROB Assessment**

A detailed description of the ROB assessment among included RCTs is presented in the diagram below.

**Summary plot of ROB for RCTs studies**
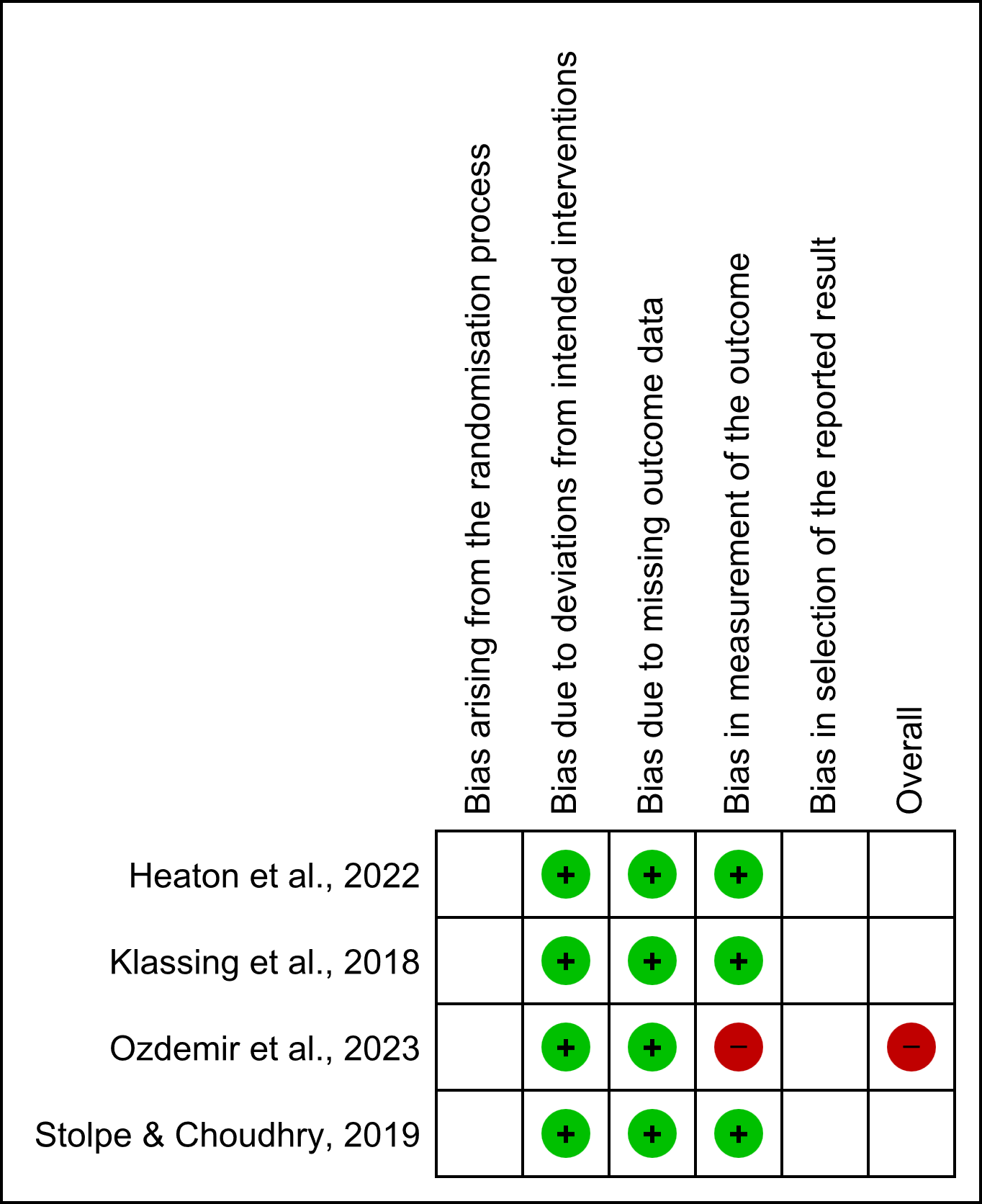

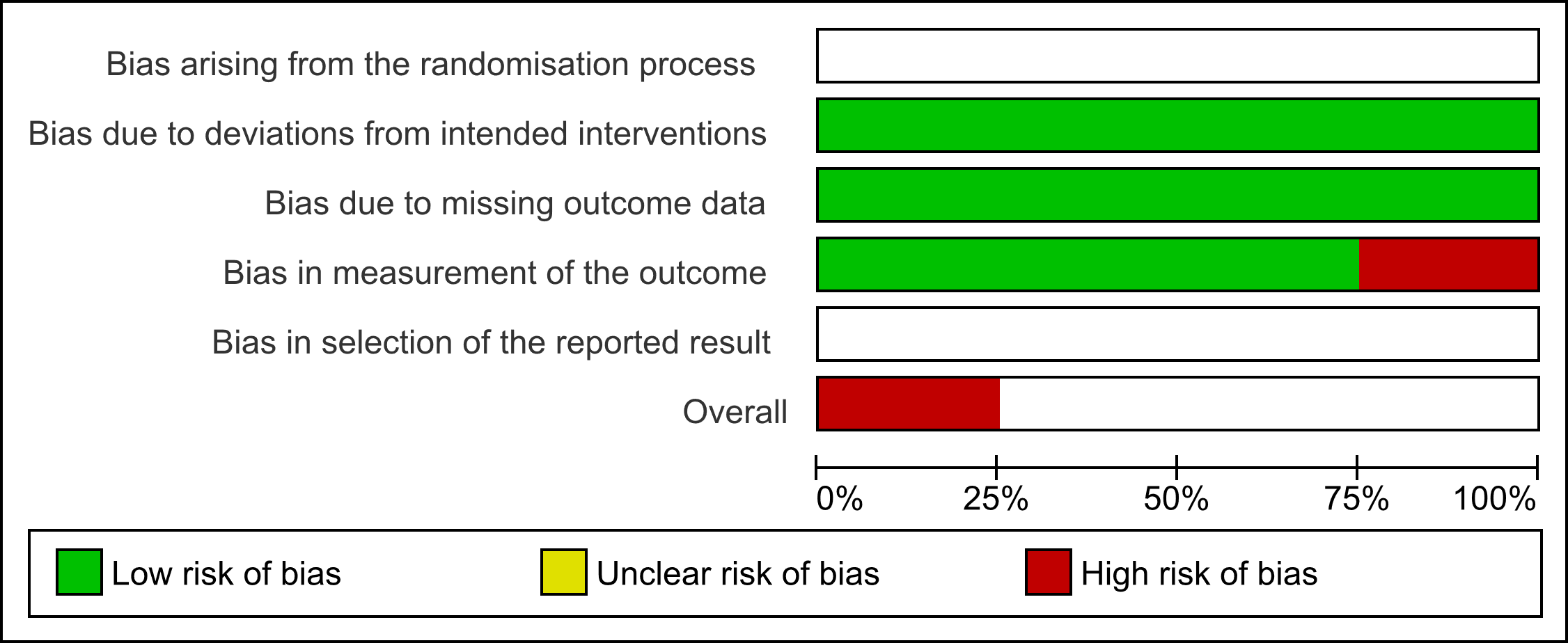


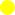

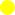

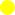

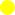

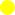

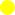

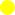

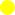

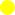

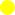

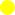


1. **Quality of Evidence from non-randomized Studies through the ROB Assessment**

A detailed description of the ROB assessment among included non-randomized studies is presented in the diagram below.

Summary plot of ROB for non-randomized studies

| **Risk of Bias Domains** | **D1** | **D2** | **D3** | **D4** | **D5** | **D6** | **D7** | **Overall** |
| --- | --- | --- | --- | --- | --- | --- | --- | --- |
| **Studies** |  |  |  |  |  |  |  |  |
| (Fathima et al., 2021) | 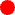 | 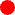 | 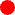 | 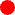 | 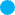 | 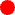 | 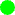 | 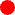 |
| (Goode et al., 2022) | 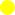 | 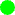 | 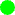 | 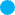 | 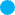 | 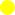 | 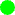 | 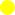 |
| (Cebollero et al., 2020) | 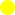 | 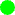 | 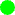 | 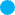 | 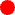 | 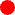 | 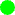 | 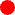 |
| (Villaverde Piñeiro et al., 2022) | 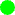 | 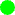 | 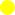 | 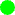 | 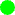 | 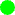 | 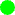 | 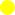 |
| (Howe et al., 2022) | 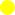 | 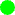 | 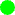 | 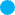 | 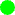 | 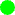 | 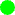 | 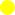 |
| (Gatwood et al., 2022) | 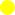 | 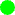 | 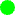 | 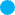 | 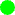 | 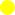 | 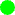 | 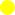 |
| (Deslandes et al., 2020) | 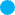 | 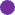 | 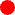 | 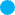 | 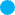 | 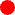 | 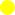 | 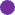 |
| (Bayraktar-Ekincioglu et al., 2022) | 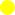 | 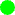 | 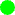 | 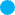 | 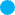 | 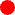 | 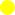 | 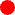 |
| (Bacci et al., 2019) | 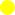 | 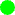 | 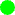 | 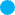 | 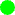 | 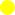 | 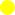 | 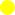 |
| (Scherrer et al., 2020) | 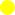 | 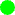 | 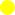 | 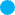 | 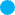 | 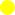 | 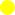 | 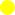 |
| (Goldsworthy et al., 2022) | 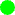 | 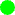 | 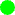 | 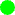 | 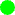 | 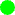 | 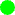 |  |
| (Abu-rish & Barakat, 2021) |  |  |  |  |  |  |  |  |
| (Page et al., 2020) |  |  |  |  |  |  |  |  |
| (Are et al., 2022) |  |  |  |  |  |  |  |  |
| (Rihtarchik et al., 2018) |  |  |  |  |  |  |  |  |
